# Many-particle Brownian and Langevin Dynamics Simulations with the Brownmove package

**DOI:** 10.1186/2046-1682-4-7

**Published:** 2011-04-13

**Authors:** Tihamér Geyer

**Affiliations:** 1 Center for Bioinformatics, Saarland University, D-66123 Saarbrücken, Germany

## Abstract

**Background:**

Brownian Dynamics (BD) is a coarse-grained implicit-solvent simulation method that is routinely used to investigate binary protein association dynamics, but due to its efficiency in handling large simulation volumes and particle numbers it is well suited to also describe many-protein scenarios as they often occur in biological cells.

**Results:**

Here we introduce our "brownmove" simulation package which was designed to handle many-particle problems with varying particle numbers and allows for a very flexible definition of rigid and flexible protein and polymer models. Both a Brownian and a Langevin dynamics (LD) propagation scheme can be used and hydrodynamic interactions are treated efficiently with our recently introduced TEA-HI ansatz [Geyer, Winter, JCP 130 (2009) 114905]. With simulations of constrained polymers and flexible models of spherical proteins we demonstrate that it is crucial to include hydrodynamics when multi-bead models are used in BD or LD simulations. Only then both the translational and the rotational diffusion coefficients and the timescales of the internal dynamics can be reproduced correctly. In the third example project we show how constant density boundary conditions [Geyer et al, JCP 120 (2004) 4573] can be used to set up a non-equilibrium simulation of diffusional transport across an array of fixed obstacles. Finally, we demonstrate how the agglomeration dynamics of multiple particles with attractive patches can be analysed conveniently with the help of a dynamic interaction network.

**Conclusions:**

Combining BD and LD propagation, fast hydrodynamics, a flexible protein model, and interfaces for "open" simulation settings, our freely available "brownmove" simulation package constitutes a new platform for coarse-grained many-particle simulations of biologically relevant diffusion and transport processes.

## Background

Before any reaction can occur in a biological cell between, for example, an enzyme and its substrate, or before two or more proteins can form a functional complex, the respective partners have to find each other in the crowded interior of the cell. For a full understanding of these association and dissociation processes, be it for a general picture or aimed at designing a drug that enhances or suppresses a certain reaction specifically, a lot of details need to be put together into a consistent picture, rate constants need to be determined, and effects of mutations need to be understood. Many of the details can be determined experimentally. Some of these information are microscopic detailed spatial pictures like crystal structures while others come from macroscopically measured data about turnovers or global reaction kinetics. All these parts of the puzzle can be assembled and studied conveniently by combining them into a computer model and performing simulations. In these *in silico *experiments all parameters of the system can then be varied to investigate their effect on the association rates and pathways. However, to deal with the large volumes and particle numbers required to describe a biological environment and the often slowly proceeding kinetics, the simulation model has to be both detailed and efficient enough. One often used workhorse technique is Brownian Dynamics, which is named after the botanist Robert Brown, who first described the microscopic random motion of pollen grains in water in a letter to his friends. Nearly eighty years later Einstein could explain his observations [1]. Brown could not see the individual water molecules in his microscope, to him it was a continuous solvent in which the visible pollen grains were floating. Einstein realised that it was the thermal motion of the water molecules which made the pollen grains move. He found that one does not have to know their individual trajectories, but that a heat bath and a Stokesian friction term are enough to describe how they push the large pollen particles around. Einstein's idea was then later cast into the Brownian Dynamics simulation technique [2]. With its continuous solvent only the trajectories of the larger particles of interest are evaluated, which allows for large simulation volumes with many particles and long simulation times. This method has been applied successfully to study, for example, bimolecular association reactions [3-6], the dynamics of colloidal suspensions and polymers [7,8], or, recently, the dynamics inside the crowded cytoplasm of a cell [9]. Einstein's ansatz to replace the solvent molecules by an effective heat bath and a friction term explains the diffusive behaviour of a single large "Brownian" particle, but it neglects all other effects of the solvent on the interactions between multiple particles. Electrostatic interactions, e.g., are shielded by the ions contained in a physiological solvent. These ions have a size which is comparable to the water molecules and thus they are normally not modelled explicitly. Their effect on the interaction between charges is usually included via Debye-Hückel continuum electrostatics. Other solvent mediated effects are the short ranged hydrophobic and hydrophilic interactions between proteins and the so-called hydrodynamic interactions which stem from the displacement of the solvent by the moving proteins, giving rise to many-particle velocity correlations.

A Brownian dynamics simulation package therefore has to deal with many different kinds of interactions, some of which are direct physical interactions while others are a consequence of the continuum solvent ansatz. For some applications like the association of two proteins, details of their spatial forms and their charge distributions are important, whereas for other applications a model of simple spheres may already capture the important features. A general purpose BD package therefore has to be very flexible and should be easily extensible.

**Figure 1:**
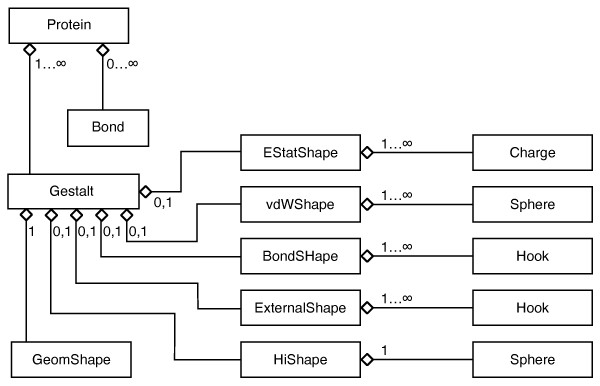
**Hierarchic construction of a general particle in brownmove**. The top level "Protein" object contains one or more "Gestalt" objects which move independently and contain a shape for each interaction, which in turn contains the basic interaction entities. These are point charges for electrostatic interactions or van-der-Waals spheres for effective short range interactions. With these the forces acting on the Gestalt are calculated. The "GeomShape" object is always present and handles the conversion of the forces into a respective displacement.

When we started with Brownian Dynamics simulations a few years ago [10,11] there was no such general purpose software for many-particle simulations available. Existing tools like UHBD, sda [12,13] or, more recently, BrownDye [14] were aimed at efficiently calculating association rates for binary encounters or, as the HYDRO suite, the diffusional properties of individual molecules [15]. We therefore developed our own package called "brownmove". The main design ideas were a modular particle model which allows to build up arbitrarily shaped particles from simple interaction shapes, and the ability to handle many-particle scenarios with varying numbers of different particles. This includes an interface to implicitly modelled bulk regions and also reactions where a particle of one type can be exchanged by a particle of a different type which, e.g., allows to describe charge transfer reactions [16]. Due to the modular particle model one can use different spatial resolutions for different particle types and it is very easy to add new types of interactions. The latest additions to the brownmove code are a Langevin propagation scheme [17] and an efficient two-body-approximation for the multi-particle hydrodynamic interactions [18]. Due to the modular design of the brownmove code these additions could be included straightforwardly. Even a mixed propagation scheme is possible, where within the same simulation the standard overdamped Brownian propagation scheme is used for one particle type while for an other type the finitely damped Langevin scheme is used. In brownmove a general particle is described by a so called "protein" object, which contains one or more "gestalt" objects which move independently. An example of multiple gestalt objects within the top level protein object could be a bead-spring-polymer, which consists of the actual beads and the springs between them. Each of the gestalt objects in turn contains one or more "shape" objects, which encode the various types of interactions. A shape for, e.g., electrostatic interactions then holds a number of point charges which can be placed at arbitrary positions within the frame of the gestalt object. This hierarchical setup as sketched in Figure 1 is implemented in C++. Each shape and the Gestalt and protein objects are classes. With this modular design, arbitrary rigid and flexible proteins can be defined and inserted into or taken out of the simulation at runtime.

While most of the interactions implemented in "brownmove" follow the usual tried strategies and approximations used in Brownian dynamics simulations of biological systems, there is only limited experience with hydrodynamic interactions (HI) in this context. The basic principles can be found in textbooks since decades, but due to the until now rather expensive numerical evaluation, simulations with HI have essentially only been performed as dedicated tests of the analytical theories while in biologically inspired projects one rather tried to get away without them.

The basic ideas of hydrodynamic interactions are simple. When a particle is moved through the solvent it drags a part of the solvent with it and thus creates a flow field that moves in the same direction as the particle. The second ingredient is Faxen's theorem which states that it takes the same force to move a particle through a solvent at rest as to keep the particle at rest in a solvent flowing around the particle with the opposite velocity. For a sphere moving with a constant velocity through a continuous solvent the flow field was calculated already by Stokes (hence the term "Stokes" friction) and can be found in most textbooks that treat fluid mechanics. A corresponding expression exists for the flow field generated by the rotation of a sphere. For translation the fluid moves in the same direction as the particle with a velocity that--to first order--decays proportional to the inverse distance. Consequently, HI are a long-range phenomenon, coupling all particles in the simulation. For accelerating particles there is, however, no closed form for the resulting flow field, because the information about any changes of the particle velocity cannot travel faster than the speed of sound, which leads to retardation effects.

In simulations with an explicit solvent the solvent molecules take care that the displacements are propagated between the particles, but in any implicit solvent method this hydrodynamic coupling has to be added back explicitly. As flow fields are associated with motion one sees that hydrodynamic interactions are not conservative forces derived from a potential energy but that they describe how the velocities of the particles influence each other. The evaluation of the actual velocity coupling is then an iterative procedure. It starts from the unperturbed, zeroth order velocities of the particles that they would have if there were no HI. From theses the resulting flow fields of the particles are evaluated and then, at the locations of the other particles, converted via Faxen's theorem into an effective force acting on them, which modifies their initial velocities. This process is iterated until convergence, which is slow due to the 1/*r*-dependence. In addition, the higher order corrections couple three, four, and more particles. For practical applications this series has to be truncated. For spheres this can be calculated analytically and results in a so-called diffusion matrix which converts the external forces acting on each of the particles into the resulting hydrodynamically correlated velocities (the mathematical details can be found, e.g., in ref. [19]). When only the first iteration is considered, this matrix is called the Oseen tensor [20]. It describes the long-range interactions, where "long-range" means that the particle diameters are much smaller than the separation. This is often expressed as that the Oseen tensor describes HI between point particles. Consequently, as points cannot rotate, there is no rotational coupling on the Oseen level. When additionally the back-coupling from the second particle back to the first is included but no three-particle terms, it is called the Rotne-Prager-Yamakawa (RPY) tensor [21,22]. This approximation is more accurate than the Oseen tensor but it still underestimates the coupling as the particles come closer. The main reasons why HI is often treated on the RPY level is that it gives reasonably accurate results for most practical applications and that for setting up this hydrodynamic tensor only pairs of particles need to be considered, which results in a runtime that scales quadratically with the number of particles. For any further orders three or more particles have to be considered. Also, rotational coupling can be included on the RPY level [23]. Higher order corrections up to 1/*r*^-7 ^can be found for example in reference [24]. Forms of the RPY tensor for spheres of different sizes can be found in [25,26].

When two spherical particles come very close the above explained iteration needs to consider impractically many terms. A more efficient approach is then to expand the hydrodynamic coupling between two particles in powers of their separation, leading to the so-called lubrication corrections (see, e.g., [27] for an application to a three-body problem).

From the above explanations one sees that HI add back the mechanical coupling between the particles that was lost in the implicit solvent approximation, albeit on a coarse and approximative level. Also, many proteins are not truly spherical and the correct hydrodynamics is different from the interaction of perfect spheres. The diffusional properties of non-spherical particles can be determined from a multi-bead-model [28,29], but there is no explicit form of the diffusion matrix for the interaction between such non-spherical objects. Here, one has to resort to a number of spheres per particle. Consequently, there has been a lot of (off the record) debate about whether the computationally expensive HI is worth the effort in BD simulations of biological scenarios where already the protein models and the interactions are modelled by crude approximations only. Due to the high numerical costs of the conventional algorithms this questions--whether and how much HI affect the dynamics in many-protein simulations--has not been addressed on a broad range. Also it is not clear yet, how much higher order corrections affect the results. With our recently presented fast TEA-HI algorithm [18], which approximates the expensive matrix factorization in the evaluation of the HI but uses the same hydrodynamic matrix, it is now possible to compare simulations with and without HI for many different scenarios. However, we were not the first ones who tried to speed up the evaluation of many-particle HI. Most prominent is Fixman's Chebyshev approximation to the expensive matrix factorization [30]. Other approaches are the Accelerated Stokesian dynamics by Banchio and Brady [31] or the mean-field-hydrodynamics of Heyes [8]. As should be clear from the above descriptions, HI on this RPY level with its multiple approximations will not be really accurate. However, a comparison between simulations with and without HI can show *whether *HI makes a difference. If the mere inclusion of RPY-HI proves critical for a certain process and one is interested in accurate quantitative results then a more elaborate method has to be used.

There is a number of methods that range in accuracy and effort between a fully atomistic model, which incorporates all details, and the simplified implicit solvent BD. One approach is to numerically solve the Navier-Stokes equation [32,33]. Other Methods like Dissipative particle dynamics [34,35], Multi-Particle Collision Dynamics [36,37], or the so-called Lowe-Anderson thermostat [38] use virtual particles to represent momentum "units" of the solvent. Yet another approach is the grid-based Lattice-Boltzmann method where a linearized Boltzmann equation is solved [39].

Another implication which has to be considered when using the simple and efficient RPY hydrodynamics in Brownian dynamics simulations are the short timescales that are required to describe the fast protein dynamics. Then, as already mentioned above, the flow fields are not the stationary Stokes solutions in an incompressible fluid anymore. On these short time and length scales an explicitly time-dependent method should be used (see for example the discussion in [40]). However, at the current stage it seems more important to map out for which types of scenarios HI does make a difference and for which it can be neglected.

This publication, which we also use to present our "brownmove" simulation package, is organised as follows. After the above introduction, the following Results and Discussions section starts with two examples on how important hydrodynamic interactions are in coarse-grained simulations of flexible proteins. The first example investigates how the stiffness of a bead-spring polymer affects its diffusional properties, while in the second example a flexible model of a compact protein is built from a number of small beads. Both cases show that hydrodynamic interactions have to be included when both rotational and translational diffusion shall be modelled correctly. In the third example non-equilibrium diffusional transport of simplified proteins through an array of fixed obstacles is simulated. The last example then demonstrates that many-particle simulations can be conveniently analysed and understood quantitatively with the help of a dynamic interaction network. The technical details of the implementation, i.e., of the propagation algorithms, the efficient hydrodynamics, the various interactions, and the available boundary conditions are given in the Methods section after the Conclusions.

## Results and Discussion

In this section we present some example scenarios to illustrate the kind of applications for which brownmove was developed. From the vast number of possible settings we chose two examples that highlight the importance of hydrodynamic interactions in coarse-grained simulations of flexible protein models and two many-particle scenarios. One describes non-equilibrium transport and the other deals with the analysis of many-particle agglomeration.

So far, two projects have been published in which the brownmove simulation package was used. These were many-particle simulations of the association of cytochrome *c *at a charged membrane [11] and a coarse-grained model of a small peptide [17]. Some other projects dealing with many-particle agglomeration and transport are currently performed. In the first project [11], the cytochrome *c *were set up with the dipolar sphere model which consists of a van-der-Waals sphere of 1.66 nm radius and three charges, a central charge of +7.5 e for the total charge of the horse-heart cytochrome *c *and two small charges of ± 1.75 e placed on opposite sides of the spherical protein to mimic the dipole [41]. The 30 × 30 nm^2 ^patch of membrane was modelled with a planar van-der-Waals surface, the charges of the lipids were implemented via a Guy-Chapman electrostatic potential plus, for some tests, nine to 25 point charges. The rectangular simulation box was only 20 nm high, so that at the highest concentrations some 300 cytochrome *c *were enough to fill up the simulation volume. The large bulk of the *in-vitro *experiments was described via our constant density interfacing algorithm, which simulates particle exchange with an infinitely large reservoir of a given density [16]. With the particle insertion interface providing a constant bulk density we determined binding isotherms which showed that even a few localised point charges on an otherwise continuously charged membrane greatly enhance the adsorption.

In the other project [17] we built a coarse-grained model of a small peptide in which each residue was set up from one to three van-der-Waals spheres and some point charges. The first van-der-Waals sphere was placed around the Cα atom. For most of the residues a second van-der-Waals sphere was enough to "cover up" the remaining part of the sidechain. Only for the largest residues, a third sphere was needed. The point charges were taken from the crystal structure and placed at the positions of the respective atoms (which generally did not coincide with the centers of the van-der-Waals spheres). Then these rigid residues were connected to their neighbours by springs between the Cα atom positions and, as required, by additional springs between residues spaced further apart. The spring constants were optimised manually against an all-atom molecular dynamics simulation. With this hand-parameterized model peptide we could show how to combine the finitely damped Langevin dynamics and the conventional Rotne-Prager hydrodynamics to yield a fast and stable propagation scheme for these small scales where BD is not applicable anymore [17]. Currently, we are working on a parameterization that allows to convert arbitrary protein structures into flexible coarse-grained models for such LD simulations.

### Example 1: HI in constrained bead-spring polymers

**Figure 2:**
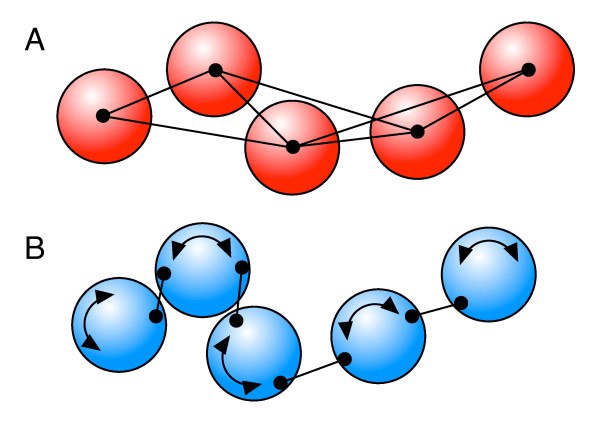
**Two ways to implement a bead-spring polymer with confined dynamics**. In the conventional variant shown in panel A the beads are connected by springs which are hooked up at their respective centers, and the bending angle between subsequent springs is confined harmonically by either a direct angle term or, as shown here, by additional springs between the next neighbours. "Brownmove" also allows to set up "bead train" polymers of rotating beads where the "front" of one bead is connected to the "back" of the previous bead with a short spring and the steric repulsion between the beads constricts the polymer dynamics. This is shown in panel B. For the simulations reported here the conventional setup A was used.

The first example presented here is a continuation of the bead-spring-polymer simulations reported in [18], which had been used to introduce and test the truncated expansion approximation hydrodynamics (TEA-HI). There, the polymers consisted of spherical beads with an exclusion volume which were connected to their direct neighbours by harmonic springs. Apart from the forbidden steric overlap between the beads there was no constraint on how these polymers might fold. In this example, we investigate the effect of polymer stiffness. As sketched in Figure 2 there are two alternative ways to introduce stiffness into the polymer chain. In the first variant the angles between subsequent springs are constrained. In "brownmove" this is done by adding additional longer springs between the next neighbours as shown in Figure 2A. This implementation was used in the project presented here. As usual in such bead-spring polymers, rotation of the spherical beads was ignored. A sample definition file for such a polymer with *N *= 5 beads and HI can be found together with the simulation setup file in the supplementary materials as additional files 1 and 2.

A second way to implement a polymer with constrained internal dynamics in "brownmove" is shown in Figure 2B. Now the beads may rotate and the springs, which only connect direct neighbours, are attached off-center. Together with the effective short-range repulsion between the beads this restricts how far the next bead may move to the side. Note that in this implementation the motion of neighbouring (and potentially non-spherical) beads is essentially free up to the point where they touch while in the first variant the bending angle is confined harmonically. In this second implementation also rotational hydrodynamics have to be considered. A brownmove example for such a bead-train polymer is given as additional file 3. However, for results which are directly comparable to the usual setups we used variant A here. Simulations with polymers of *N *= 3, 5, 10, and 15 beads, respectively, were run both with and without Rotne-Prager hydrodynamics using our TEA-HI algorithm. The radius and the translational diffusion coefficient *D*_bead _of the beads was set to 1, which required a simulation timestep of Δt = 5 × 10^-4^. The springs between the direct neighbours had a spring constant of *k*_N _= 1000 units and the stiffness of the angle confining next neighbour springs of length 5 was varied from *k*_2N _= 0.1 to 1000. The springs between the direct neighbours had a length of 2.5 such that neighbouring beads did not overlap. Overlap between all other pairs of beads was prevented by a short range repulsive potential.

**Figure 3:**
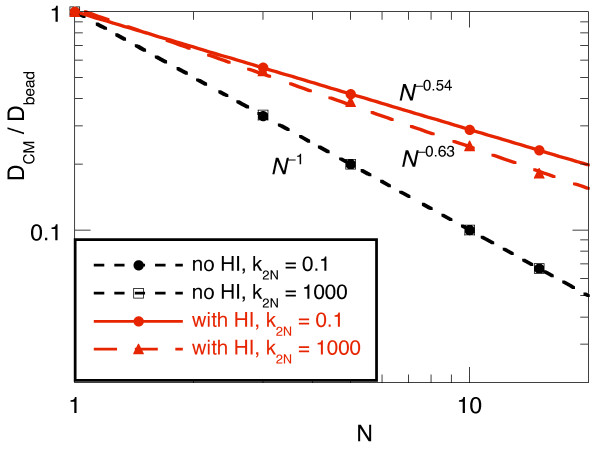
**Normalised center-of-mass diffusion coefficient *D*_CM _of bead-spring polymers vs. the number of beads, *N***. Without HI, *D*_CM _scaled as *N*^-1 ^independent of the stiffness, i.e., the "folding" state of the polymer. With HI, a stiffer polymer with a higher *k*_2N _diffuses slower because then the beads are on average further away from each other and the velocity correlation introduced by the HI is weaker.

Figure 3 shows how the long-time center-of-mass diffusion coefficient *D*_CM _of the polymers scaled with their bead number *N*. Without hydrodynamics, *D*_CM _scaled as *N*^-1 ^independent of how flexible the polymer was. With HI and very soft springs our polymer resembles the conventional unconstrained model and, correspondingly, the scaling of *D*_CM _was close to the theoretical prediction of *N*^-0.588 ^for infinitely long polymers [7,42]. When the stiffness of the polymer is increased the beads are further away from each other on average and the effects of hydrodynamics decrease and, correspondingly, *D*_CM _decreased faster with *N *when *k*_2N _was increased. This effect is not very pronounced for the short polymers investigated here. Therefore, only the lowest and highest values of *k*_2N _= 0.1 and 1000 used here are shown in Figure 3. For even higher spring constants *k*_N _and *k*_2N_, the polymers would start to resemble long rods for which *D*_CM _scales as ln(*N*)/*N *[19]. Indeed, some deviations from the power-law scaling can already be seen for *k*_2N _= 1000.

**Figure 4:**
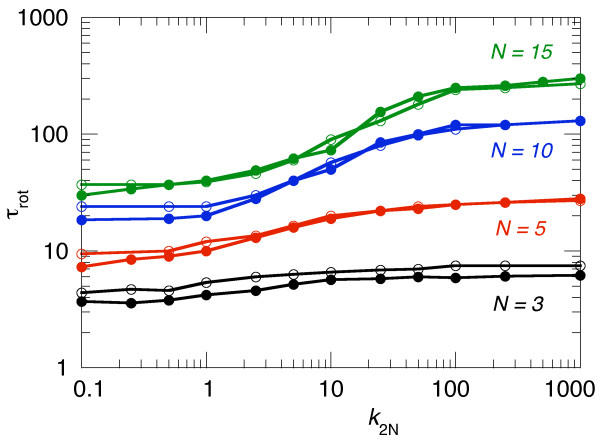
**Correlation time **τ**_rot _of the orientation of the end-to-end vector vs. polymer stiffness**. When the spring constant of the next neighbour springs, *k*_2N_, was increased the polymer became more extended and, consequently, rotated slower. The filled symbols are from simulations without HI while the open symbols give the results with HI for polymers of the respective bead number *N*.

A different behaviour was found when the rotational properties of the polymers were considered. As shown in Figure 4, the correlation time τ_rot _of the orientation of the end-to-end vector was nearly insensitive to whether HI was included or not. However, now the stiffness, which determines the average length of the polymer, had a strong effect on rotation. Whereas for the short polymer with *N *= 3 the length, and therefore τ_rot_, could not vary much, the orientational relaxation time increased by about one order of magnitude for the long *N *= 15 polymer when *k*_2N _was increased from 0.1 to 1000.

From these observations the following picture emerges. Translational diffusion of our polymers was only weakly affected when the more globular structure of a flexible polymer was "unfolded" by increasing the chain stiffness. However, it was crucial to include HI for the correct scaling of *D*_CM _with the chain length. This is even more important when proteins are simulated which can fold into a much more compact structure than these bead-spring polymers with only repulsive interactions between their beads. On the other hand, the rotational motion was very sensitive to the "folding state" of the polymer but essentially unaffected by HI. Thus, if one wants to use a bead-spring polymer as a model for a protein which can change its conformation from a folded globular to a denatured unfolded state, then HI has to be included. Otherwise, only the translational *or *the rotational diffusional behaviour can be modelled correctly, but not both.

In these bead-spring polymers the direct interactions between the beads, i.e., the springs and the van-der-Waals interactions, were very simple and thus the relative costs of including the hydrodynamic coupling were relatively high. On average, the simulations with HI took about three times as long as the corresponding simulations without HI. However, due to the O(*N*^2^) scaling of our TEA-HI algorithm this ratio was roughly constant for all polymer lengths and thus one can state that when a simulation can be afforded without HI, then normally the corresponding scenario with HI can now be done, too.

### Example 2: Elastic proteins from small beads

**Figure 5:**
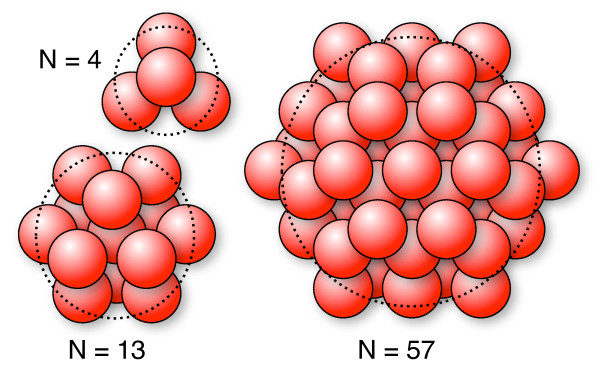
**Sketch of three of the elastic protein models assembled from hexagonally placed small beads**. The beads are held together by harmonic springs between direct neighbours which are then propagated individually. In addition to the three sizes shown here we used a fourth protein built from 39 beads. As more and more beads are used the resulting particles get closer to a spherical shape.

The previous example shows that it is crucial to include HI when the diffusional properties of polymers or proteins are simulated with bead-spring models. The following example confirms this finding. For this we built proteins from small beads which interact via HI as pioneered by García de la Torre (see, e.g., [15,28,43]). For easier comparison we focussed here on (nearly) spherical shapes. The particles were assembled from beads placed onto a hexagonal close packing grid as sketched in Figure 5. Each bead had a radius of 2 nm and a corresponding single bead diffusion coefficient of *D*_bead _= 1.2 × 10^-4 ^nm^2 ^ps^-1^. The beads were connected to their up to twelve direct neighbours by springs with a length of 5 nm. Four different particles were assembled with *N *= 4, 13, 39, and 57 beads, respectively. Three of them are sketched in Figure 5. As an example the definition file for the *N *= 13 particle with HI is given as additional file 4.

**Figure 6:**
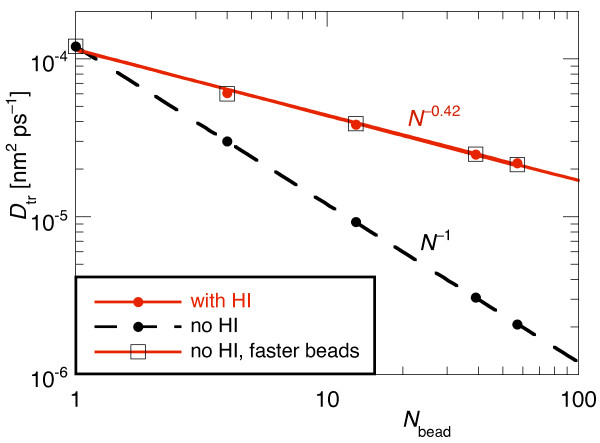
**Scaling of the translational center-of-mass diffusion coefficient *D*_tr _of spherical multi-bead particles with the number of beads, *N***. Without HI the usual *N*^-1 ^scaling was obtained whereas with HI the scaling was close to the *N*^-1/3 ^scaling for a single sphere when the volume is proportional to the number of beads. The open squares denote results from simulations without HI in which the single bead diffusion coefficient was rescaled for each particle size (= bead number *N*) individually in order to reproduce the scaling of *D*_tr _with HI.

From the simulations we extracted the center-of-mass diffusion coefficient *D*_tr_. As shown in Figure 6, *D*_tr _again scaled as *N*^-1 ^when HI were neglected. This is the same scaling as for the polymers which have a completely different shape. With HI included, *D*_tr _decreased as *N*^-0.42 ^with the number of beads. This is slower than in the polymer case because here the particles are much closer together on average than in the flexible, chain shaped polymers.

For comparison, the translational diffusion coefficient of a sphere scales proportional to its inverse radius [19]. Assuming a constant density, we find that *D*_tr _decreases with the third root of the volume. We can thus estimate that in our case where the volume is proportional to the number of beads, *N*, *D*_tr _should decrease as *N*^-0.33^. Our results were slightly slower because both the Rotne-Prager tensor and our TEA-HI algorithm underestimate the hydrodynamic coupling at close distances [18,24]. Also, we did not investigate how the radius of the individual beads affects the diffusion coefficients of the assembled proteins (see, e.g., the discussion in [44]).

When a protein is modelled from individual beads one can adjust the diffusion coefficient *D*_bead _of the individual beads until a best match between an experimentally determined diffusion coefficient and the *D*_tr _observed in the simulation is achieved. Here we could not compare to experimental values but we ran another set of simulations without HI where *D*_bead _was adjusted such that the center-of-mass diffusion reproduced the values obtained with HI. The required single bead diffusion coefficients were 2.4 × 10^-4^, 5 × 10^-4^, 9.7 × 10^-4^, and 1.2 × 10^-3 ^nm^2 ^ps^-1 ^for the particles with N = 4, 13, 39, and 57 beads, respectively. Consequently, in Figure 6 the obtained *D*_tr _values with the faster beads coincide with the results with HI.

**Figure 7:**
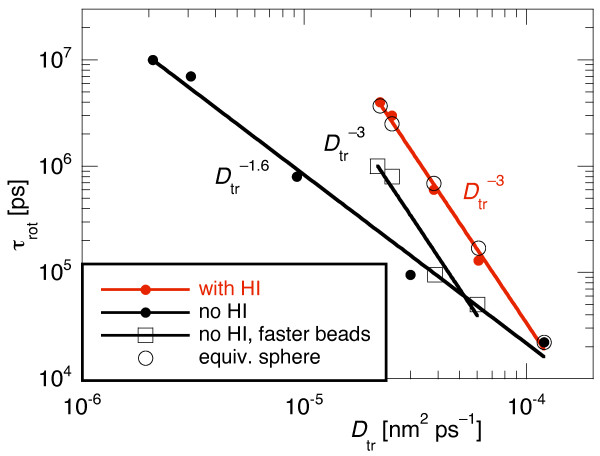
**Rotational relaxation time of the end-to-end vector, t_rot_, vs. the center-of-mass diffusion coefficient *D*_tr _for multi-bead particles of different sizes *N***. With HI the expected  scaling of a sphere of equivalent radius was reproduced, whereas without HI rotation was too fast. When the single bead diffusion coefficients were rescaled for the correct *D*_tr _at each *N *individually (open squares), τ_rot _also scaled as  but was still too fast by a factor of about five. For more explanations see text.

The rotational diffusion coefficient *D*_rot _= k_B_T/8πη*a*^3 ^of a sphere of radius *a *decreases linearly with the volume. Together with the *N*^-1/3 ^behaviour of *D*_tr _we consequently find that the rotational relaxation time τ_rot _= 1/2*D*_rot _scales proportional to *D*_tr_^-3 ^for a spherical particle. Figure 7 shows that this prediction was indeed fulfilled by our "spherical" particles assembled from individual beads when HI were included. Without the hydrodynamic velocity coupling between the beads, τ_rot _only scaled as  which means that the rotational relaxation was too fast compared to the translational diffusion. A large particle built from many small beads thus either rotates too fast or translates too slow. Interestingly, the correct  scaling was regained when the single bead diffusion coefficient *D*_bead _was modified as explained above such that for each particle size the correct *D*_tr _was obtained. Then, however, rotation was about five times too fast for all our test particles. This again shows that it is essential to include HI in multiple-bead models of, e.g. proteins in order to correctly describe *both *the translational and the rotational diffusion (see also [44]). On the other hand, with HI, the correct rotational diffusion comes for free in multi-bead models. As shown once more, multi-bead models can be used to correctly describe both translational and rotational diffusion for arbitrarily shaped proteins. This could have also been achieved with much less numerical effort with a rigid particle and a non-isotropic diffusion matrix, which can be determined off-line from a bead model [15].

However, as shown by Elcock *et al*. [44], a multi-bead model "automatically" yields a much more reliable description of the hydrodynamic interactions between different particles. Additionally, the Rotne-Prager tensor is a far-field approximation which works reasonably well when two spherical beads are separated by more than a radius inbetween them. With the smaller beads of a multi-bead model HI thus becomes more accurate for smaller separations between the particles.

Another reason to use multi-bead models is that they allow to describe the internal flexibility of a protein, where often conformational changes occur upon binding or during the catalytic activity or, most prominently, when a protein folds. In these scenarios it would be a bad idea to rescale the single bead diffusion coefficients because even though the overall diffusive behaviour might be described better, the internal dynamics would take place on the too fast timescale of the individual beads. Our "spherical" particles do not undergo conformational changes but it is nevertheless interesting to investigate the dynamics of the individual beads. At very short timescales the elastic springs do not affect the small thermal fluctuations of the beads whereas for very long observation intervals the beads are effectively frozen to their positions in the particle. We thus expect that *D*_bead _extracted from a simulation decreases with increasing observation interval from the single bead value *D*_bead_(Δt) to the center-of-mass *D*_tr_.

**Figure 8:**
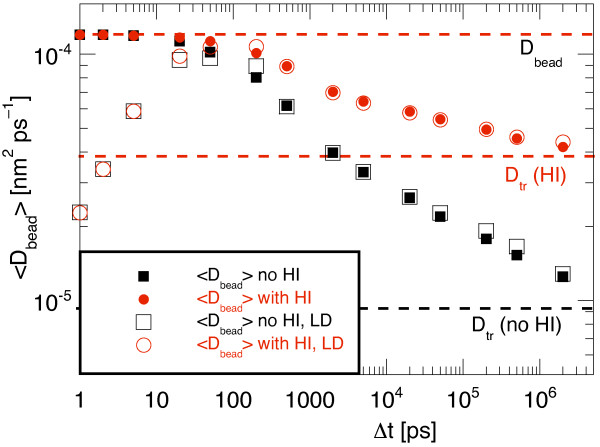
**Effective average diffusion coefficient <*D*_bead_> of the individual beads in a multi-bead model vs. the length of the observation interval Δ*t***. With BD the dynamics of the mass-less beads is determined on short time scales by the fast thermal motion of the individual beads while over longer periods the beads have to follow the center-of-mass motion of the complete particle (filled symbols). Consequently, over long intervals Δ*t *the effective average bead diffusion coefficient <*D*_bead_>(Δ*t*) converges to *D*_tr_. Without HI the two underlying timescales are separated even further. When the mass of the beads is considered and an LD propagation is used then the apparent <*D*_bead_> also decreases at very short observation intervals independent of whether HI are used or not.

For this we determined the apparent single bead diffusion coefficients *D*_bead _(Δt) = <*r*^2^(Δ*t*)>/6Δ*t *of all individual beads of the *N *= 11 particle from the averaged squared displacements <*r*^2^(Δ*t*)> for different observation time intervals Δ*t*. This analysis was performed on a simulation where the mass of the beads was neglected, i.e., with the BD propagation scheme, and on another similar simulation using the finitely damped LD scheme with a bead mass *m*_bead _= 20 kDa. The corresponding velocity relaxation time is then τ_rel _= 0.99 ps which is about the length of the shortest analysis timestep used here. The filled symbols in Figure 8 show how with the BD propagation the ensemble average <*D*_bead_>(Δ*t*) decreased from the single bead value of 1.2 × 10^-4 ^nm^2 ^ps^-1 ^for very short intervals to the long-time center-of-mass value *D*_tr_. The behaviour of the apparent <*D*_bead_>(Δ*t*) was similar both with and without HI, but the difference was larger without HI. This again illustrates that when *D*_bead _is rescaled to compensate for the omission of HI in such a multi-bead model the internal dynamics will be too fast in relation to the diffusional center-of-mass displacement. One can expect that then also smaller domains of a few connected beads move too fast in comparison to larger domains. This may affect for example the folding trajectories of proteins or the relative ordering in a sequence of conformational changes of a protein as demonstrated in [45]. When the mass of the beads was considered (open symbols in Figure 8) the same long time values were obtained, whereas <*D*_bead_> dramatically decreased when. Δ*t *was made smaller. At Δ*t = *1 ps ≈ τ_rel _the apparent <*D*_bead_> = 2.3 × 10^-5 ^nm^2 ^ps^-1 ^was nearly one order of magnitude smaller than the specified long time value of *D*_bead _= 1.2 × 10^-4 ^nm^2 ^ps^-1^. It is interesting to note that this slowing down of the short-time dynamics was essentially unaffected by HI. Apparently, in a BD or LD simulation the hydrodynamic velocity coupling requires some time to build up before it affects the relative motion of the individual beads.

Comparing the runtimes for the various protein sizes we again find that the effort per timestep scales quadratically with the number of beads, *N*, both when HI are included and when not. With HI, the simulations took about three times as long as without. From these runtimes we can estimate that up to a million timesteps of a many-particle simulation with a hundred simple rigid particles or individual shapes can be computed in about one hour on one core of a 2 GHz Core2 Duo CPU. With a typical timestep of 10 ps for a many-protein scenario this amounts to a total simulated time of 10 μs per hour.

Here, however, a word of caution is required. From a theoretical point of view it is not correct to use RPY hydrodynamics which are based on stationary flow fields together with the LD propagation algorithm with its acceleration phases. Here an explicitly time dependent ansatz for HI should be used [40]. Thus, using BD with HI seems to be the better choice regarding theoretical consistency--but using BD for fast processes is questionable, too. To add to this uncertainty, it is also not clear how one should interpret *hydro*dynamic interactions inside a compact protein where there are usually only a few scattered water molecules? Yet another view to this problem would be to observe that for very short Δ*t *HI effectively become irrelevant for both BD *and *LD and to conclude from that that for practical applications HI should just be used. They are required for the long time dynamics and do not hurt the details.

Probably, one should use a different interpretation for *inter*- and for *intra*-molecular HI in simulations as presented above. The *inter*-molecular HI describe the solvent-mediated velocity coupling via the resulting flow fields and is thus the "true" HI whereas the *intra*-molecular HI recover a part of the internal viscosity of the protein: when a multi-bead-model is used to coarse-grain a protein then each bead is a rigid representation of a part of the originally continuously elastic protein. Instead of the continuous deformations of the original protein, now these rigid blocks move relative to each other and HI may be a way to recover at least a part of the viscosity of the protein. Consequently, with all these uncertainties in mind, we find that some more research is required in this area of elastic coarse-grained protein models and fast algorithms for time-dependent HI to finally arrive at both a sufficiently accurate mathematical formulation and the correct interpretation.

### Example 3: Diffusional transport around fixed obstacles

**Figure 9:**
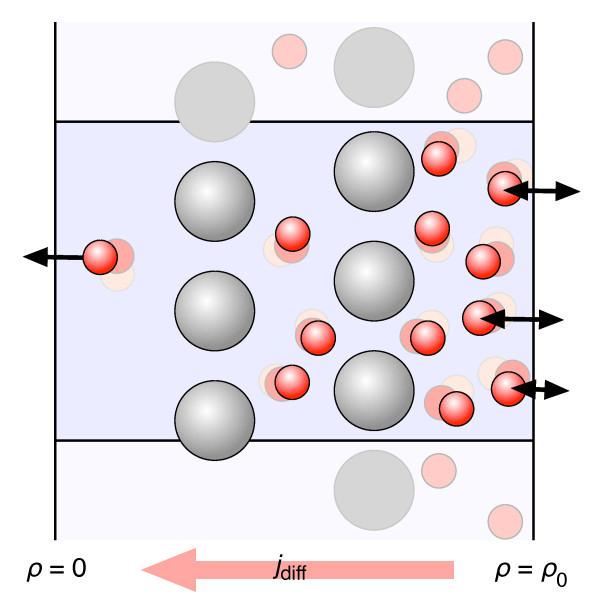
**Diffusional transport through an array of fixed obstacles**. The 2D periodic simulation box is bounded in the third dimension by two reservoirs with fixed densities ρ = ρ_0 _and ρ = 0 between which a stationary diffusion current develops. This current depends on the number and size of the fixed spherical obstacles (grey) and on their interaction with the small mobile particles (red). For more details see text.

The third example presented here relates to diffusional transport in a cell where many fixed obstacles like the cytosceleton or small vesicles obstruct the free diffusion of the soluble proteins. Our (non-equilibrium) setup is sketched in Figure 9. A rectangular simulation box of length *L*_x _= 30 nm and area *L*_y_*L*_z _= (20 nm)^2 ^was placed between two reservoirs with fixed densities ρ, of which one was set to a finite value ρ_0 _= 4 × 10^-4 ^nm^-3 ^= 0.67 mM and the other to ρ = 0. After an equilibration phase a constant diffusion current developed which depends on the density difference ρ_0 _and on the number and size of the fixed obstacles in the simulation volume. Here, we used one or two layers of nine obstacles in the (2D periodic) y-z-plane. The diffusing "proteins" had a radius of *a *= 2 nm and a diffusion coefficient of *D*_0 _= 10^-4 ^nm^2 ^ps^-1^. They were uncharged and their van-der-Waals shapes prevented a mutual overlap. The obstacles were placed on a 3 × 3 rectangular grid either at *x *= 0 when one layer was used or at *x *= ± 10 nm with two layers. With this setup the obstacle layer became impermeable for the proteins when the obstacle radius was larger than *A*_o _= 7.4 nm. More details can be found in the actual setup and particle definition files given as additional files 5, 6, and 7.

In our "brownmove" simulations the obstacles were implemented as a "Wall" object with a "Gestalt" that consisted of nine (18) van-der-Waals spheres at fixed positions. As these fixed structures do not move anyway, no mutual interactions between them are evaluated, so that the number of pairwise forces *N*_int _that has to be determined at each timestep is given by(1)

where *N*_p _is the number of mobile particles and *N*_o _is the number of fixed obstacles. This means that for larger systems the runtime still scales with  instead of *O*((*N*_p _+ *N*_o_)^2^) if the obstacles were implemented as mobile particles that are confined to their locations by, e.g., harmonic constraints. One caveat though is that in "brownmove" hydrodynamic interactions between moving proteins and fixed obstacles are not implemented yet. Consequently, in this project no HI was used. With non-interacting particles and no obstacles in the simulation volume the resulting diffusion current *j*_D _= *D*_0 _ρ_0 _/*L*_x _would be *j*_D _= 6.7 × 10^-10 ^ps^-1 ^nm^-2^, i.e., particles would arrive at the ρ = 0 boundary with a rate of *R*_D _= *L*_y_*L*_z_*j*_D _= 1.1 × 10^-6 ^ps^-1^. With our finite sized particles we obtained *R*_D _= 1.2 × 10^-6 ^ps^-1 ^from a control simulation without obstacles. All simulations were run for 10 million timesteps of Δ*t *= 10 ps with initially no mobile proteins in the simulation volume. Each simulation took about half an hour on one core of a 2 GHz Core2 Duo CPU. During each simulation we counted the number of particles *N*_L_(*t*) that left the simulation at the (left) ρ = 0 interface. Three simulation runs were performed for each configuration and the averaged *N*_L_(*t*) was fitted with a straight line. Its slope gave the diffusion rate *R*_D _through the simulation volume with the respective array of obstacles and the intersect with the x-axis corresponds to the time *T*_D _that the particles needed to cover the distance *L*_x _around the obstacles. The results are given in table 1 which also lists the average number *N*_p _of mobile particles in each of the simulations. The investigated configurations were a single or a double layer of obstacles with radii *A*_o _= 5 to 7.3 nm and either purely repulsive short range interactions between all particles or with an additional attractive term between the obstacles and the diffusing proteins.

**Table 1:** Diffusive particle transport through an array of fixed obstacles.

Setup	*N*p	*R*_D _[μs^-1^]	*T*_D _[μs]
no obstacles (control)	20	1.2	8
single layer, repulsive, *A*_o _= 5 nm	17	0.69	8
double layer, repulsive, *A*_0 _= 5 nm	15	0.55	12
double layer, repulsive, *A*_0 _= 6 nm	11	0.16	10
single layer, attractive, *A*_o _= 5 nm	22	1.2	5
double layer, attractive, *A*_0 _= 5 nm	25	1.2	5.5
double layer, attractive, *A*_0 _= 6 nm	25	1.0	6
double layer, attractive, *A*_0 _= 7.3 nm	27	0.72	7

Number of particles in the simulation volume, *N*_p_, rate of particles that crossed the simulation volume, *R*_D_, and the time *T*_D _that the particles needed to diffuse through the simulation box for the different setups that were simulated.

The results in table 1 show that with purely repulsive interactions the number of particles in the simulation volume, *N*_p_, and the diffusion rate *R*_D _decreased as expected with each additional layer of obstacles and also with the obstacle radius *A*_0_. It also took the particles slightly longer to travel through the simulation volume when the second layer was added because now the shorter direct path was blocked and the particles had to diffuse around the obstacles. When the obstacle radius was increased beyond *A*_0 _≈ 6.5 nm the diffusion current was blocked within the scope of our simulation. Then also *T*_D _become very large and no particles reached the ρ = 0 side within the given simulation duration of 100 μs. Intrestingly when a short range attractive interaction was added between the proteins and the obstacles, diffusion was much less hindered. Even two layers of obstacles with *A*_0 _= 5 nm did not reduce the diffusion rate *R*_D_. Due to the attraction the proteins stayed closer to the obstacles which reduced the effective "bulk" density. Then, more particles were inserted and *N*_p _increased. When the proteins are attracted to the obstacles they temporarily slide along their surfaces and are thus effectively funnelled through the gaps between the obstacles. Consequently, it took them less time to pass the obstacle "barrier" and *T*_D _decreased considerably. To shut off the particle transport the now attractive obstacles had to be made so large that the proteins did not fit through the pores anymore. This occurred at *A*_o _≥ 7.4 nm. The efficiency of the surface-induced funneling was so high that even at *A*_0 _= 7.3 nm, where the pores between the obstacles were only slightly larger than the proteins, the diffusion rate was about as high as with a single layer of much smaller repulsive obstacles.

In this simplified scenario both the mobile "proteins" and the obstacles were perfect spheres without any surface roughness and without hydrodynamic interactions which would slow down the protein diffusion close to the large obstacles [46]. In a more realistic setup one would therefore expect that an unspecific attraction makes diffusion between fixed obstacles faster while a corrugate surface would introduce sticking and thus break the surface induced funnelling observed here. Then also HI should be included by implementing the obstacles as mobile proteins that are fixed to their positions by harmonic external potentials. However, such a detailed project with realistic shapes and interactions is beyond the scope of this publication but it can be implemented straightforwardly in brownmove based on the this example.

### Example 4: Particle agglomeration networks

**Figure 10:**
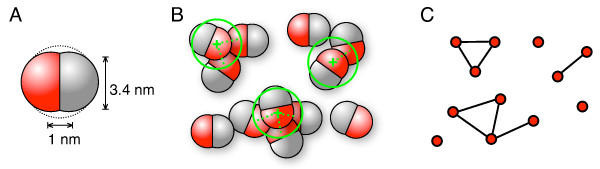
**Simulation of particle agglomeration and analysis via a dynamic network**. Sketch of the particles composed of two mutually displaced van-der-Waals spheres (A). As indicated in B, the particles can bind to each other with their red sides. The spatial snapshots are mapped onto an interaction network by using a distance criterion. The resulting dynamic network is then used to analyse the simulation (C).

In the last example we present simulations of the agglomeration of simple particles with a binding patch and how such many-particle simulations can be analysed conveniently with the help of a dynamic interaction network. This idea was previously introduced in reference [47]. For an introduction into networks in a biological context see for example [48]. As sketched in Figure 10 A the particles were composed from two van-der-Waals spheres of 1.7 nm radius which were displaced from the particle center by ± 0.5 nm in opposite directions. Each half had a different van-der-Waals "colour", as this index is named in "brownmove". Now for each pair of colours a set of interaction parameters was defined such that the red spheres of Figure 10 interact with a repulsive hard core plus a short ranged attractive term and the other combinations -- red against grey and grey vs. grey -- had a repulsive interaction only. The well depth of the attractive potential between the red spheres was slightly below the thermal energy so that in the simulations complexes formed only transiently. Each particle had the translational and rotational diffusion coefficients of an equivalent sphere of 2 nm radius, i.e., *D*_tr _= 1.2 × 10^-4 ^nm^2 ^ps^-1 ^and *D*_rot _= 2.26 × 10^-5 ^ps^-1^. With the mass of a protein of that size of *m *= 18 kDa we get a velocity relaxation time *m*/γ = 0.9 ps. Even though a relatively large timestep of Δ*t *= 10 ps was used in the simulations, we ran them with the LD propagation scheme because its overhead is negligible and the propagation is more stable than the standard BD scheme [17]. This is especially useful in such many particle agglomeration scenarios where large forces may occur locally. In such cases the finitely damped LD scheme has an additional safety margin.

The simulation was performed with 27 particles in a cubic simulation box of 30 nm length with 3D periodic boundary conditions for 20 μs. Every 10 ns the positions and orientations of the particles were saved to disk. The particle definition and the brownmove simulation setup are given as additional files 8 and 9. After the simulation, which took about 35 minutes on one core of a 2 GHz Core2 Duo CPU, the positions of the red vdW spheres were extracted from the trajectory dump and for each timestep an interaction network was constructed with a distance criterion as indicated in Figure 10B and 10C. For this, each red vdW sphere corresponds to a node of the network and a link was added between all nodes that had a center-to-center distance of less than 4 nm. A spatial configuration as shown in Figure 10B where some of the distance checks are indicated by the green circles would then result in a network as shown in panel C. For illustration purpose the nodes of the network are placed at similar positions as the corresponding vdW spheres, but for our actual network analysis the spatial coordinates were not used anymore once the network was set up. From the dynamic network then typical network measures like the total number of links or the size distribution of the clusters were extracted at each output step.

**Figure 11:**
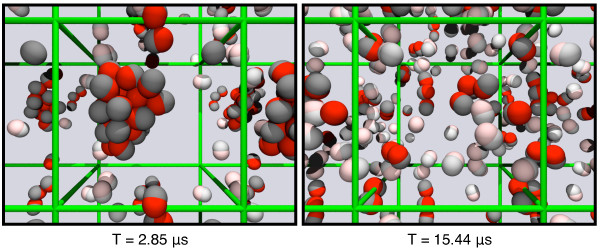
**Two snapshots of a simulation of particle agglomeration**. At *T *= 2.85 μs most of the particles were found together in one large cluster, while later at *T *= 15.44 μs the largest cluster consisted of three particles. The time points of these two snapshots are indicated in figure 12 A with black arrows.

To demonstrate the convenience of such a network analysis we first present in Figure 11 two snapshots from the simulation (no further snapshots nor a movie will be given). They show the particles with their periodic images in the x-y plane. For clarity, the periodicity in z-direction is omitted. The left panel shows the simulation at *T *= 2.85 μs when a large cluster of 21 particles and a dimer had formed. The remaining four particles were unbound and are shown in the image in lighter colour. Later, at *T *= 15.44 μs as shown in the right image, more than half of the particles were not bound to any other and the largest cluster had a size of three. To explain the association and dissociation events during the complete simulation, one would normally generate a movie and observe how quantities like the total binding energy or the radial correlation function evolve over time.

**Figure 12:**
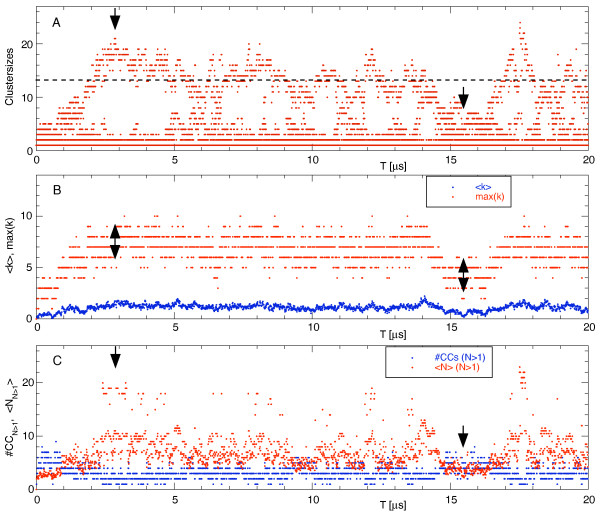
**Network analysis of a particle agglomeration simulation**. Panel A shows which cluster sizes occurred during the simulation. The two arrows denote the time points of the two snapshots shown in figure 11. The other two panels give the average and the maximal degree, <*k*> and max(*k*), respectively, (B), and the number of clusters of size larger than one, #CC(*N*>1), and the average size of these cluster, <*N*>(*N*>1) (C).

Using the dynamic network it is now actually quite easy to visualise the clustering dynamics quantitatively. Figure 12A shows which cluster sizes occurred during the simulation. A dot denotes that at least one cluster of the given size was found in the simulation at that time. One sees that in the beginning small clusters of up to 6 particles formed quickly and that after the first microsecond one cluster grew until its size peaked at 21 particles in the snapshot shown above (indicated by the black arrow). The broken line indicates the half of the 27 particles. Thus, when a cluster with at least 14 particles occurs we can be sure that there is only one large cluster whereas for cluster sizes of one or two there were usually a few clusters at the same time in the simulation. The broad distribution of the cluster sizes shows that the clusters were highly dynamic with many fast binding and unbinding events which took place on timescales faster than the size of the dots in Figure 12. After the largest cluster had its maximum size around *T *= 2.85 μs, it started to slowly shrink again. Around *T *= 6 μs we can see a broad band of cluster sizes of up to 14 which indicates that the largest cluster was falling apart temporarily into two or more smaller parts and then re-stabilised again after *T *≈ 7 μs. The second snapshot shown above is from the region of *T *= 15...17 μs when there was no large cluster. Interestingly, after this "reorganisation" phase, another large cluster formed quickly which contained nearly all particles around *T *= 17.5 μs.

Panel B of Figure 12 gives the average degree <*k*>, i.e., the average number of links per node, and the maximal degree during the same simulation run, max(*k*). To a first approximation each link contributes the same amount to the total binding energy of the system. This panel therefore can be compared to a plot of the total energy of the simulation in a conventional spatial analysis. We see that except for the beginning of the simulation and during the "reorganisation phase" *T *= 15...17 μs both the averaged <*k*> and the maximal degree only fluctuated around their typical values and especially the formation of the large clusters cannot be identified from this plot. This can be understood because with the many independent fast binding and unbinding events between the particles each particle will on average have a similar number of binding partners and the maximal degree is limited by the size of the neighbouring particles. Thus only a certain number of particles can come close enough to a given particle to be counted as bound. However, from the comparison of panels A and B we find that the large clusters identified in the cluster size distribution were not very compact. Otherwise, <*k*> and max(*k*) would peak at the same time points as the cluster size.

A third view onto the simulation is presented in panel C which gives the number of clusters (connected components) of sizes larger than a single particle, #CC(*N*>1), and the average size of the clusters of at least two particles, <*N*>(*N*>1). These two graph measures again indicate that the simulation was highly dynamic. The number of clusters of at least two connected particles fluctuated between one and about five, indicating that quite often during the simulation there was only one large cluster plus a number of unconnected particles. These events coincide with a large average cluster size. In fact, when there is only one large cluster then the average size is the same as its size.

In general we find that the more local measures such as the degree of a node give less information about the overall state of the simulation than the more global ones like the cluster size distribution. Other helpful measures which were not presented here include the clustering coefficient which quantifies how well or how regular the neighbourhood of a given particle is connected, or the distribution of shortest paths which gives a measure about how densely packed the clusters are.

## Conclusions

In this publication we gave four simple examples for simulation projects that can be performed "out of the box" with our brownmove simulation package. The main features presented here are the fast hydrodynamics algorithm, the easy and flexible setup of mobile proteins and of fixed structures in the simulation volume, and how a dynamic network can be used to conveniently analyse a many particle simulation quantitatively and to visualise the fast changes of the time dependent spatial properties.

In the first example we investigated how the stiffness of a bead-spring polymer which was controlled by additional springs between next neighbours, affects the translational and rotational diffusion coefficients. This model system can be compared to a denatured protein in unfolded states ranging from a molten globule like structure of the completely flexible polymer up to a rather rod-like structure when the 1-3-springs are made stiffer. In experiments one finds that a more stretched configuration generally diffuses slower. When hydrodynamic interactions (HI) are omitted--as was often done in BD simulations of protein--the long-time translational diffusion coefficient was completely insensitive to the "folding state" of the polymer. Only when HI were included we observed that the more stretched stiffer polymers diffused slower than the more compact flexible versions. Interestingly, the rotational diffusion, characterised by the relaxation time τ_rot _of the end-to-end vector, was only very weakly affected by HI. While the (constrained) bead-spring polymer can be interpreted as an unfolded protein, the flexible particles of our second example represent folded proteins. They were built from different numbers of small beads placed on a hexagonal close packing lattice and connected by springs to their direct neighbours. Again we examined translational and rotational diffusion and compared our results to the predictions for a sphere with an equivalent radius. In the simulations with HI the predicted scaling both of the center-of-mass diffusion coefficient *D*_tr _and of the orientational relaxation time τ_rot _with the number of beads was observed, whereas without HI we could only reproduce either *D*_tr _or τ_rot _by rescaling the diffusion coefficients of the individual beads, but not both at the same time. Generally, without HI either rotation was too fast or translation too slow. From these two examples one finds that HI must be included in simulations of flexible protein models when translation, rotation, and internal dynamics are investigated. With HI, the translational diffusion coefficient of the individual beads is then the only parameter that needs to be adjusted. The rotational properties and also the relative timing of internal motions then come "for free". This was already briefly discussed recently by Frembgen-Kesner and Elcock [44]. Whereas there still the usual numerically expensive Cholesky factorisation was used to evaluate the hydrodynamic correlations, we used the recently introduced truncated expansion approximation with its much faster O(*N*^2^) scaling. For these two examples with their very simple beads the inclusion of HI only slowed down the simulations by a factor of about three. When the individual sub units are more complex, i.e., when additionally point charges are included or more than a single van-der-Waals sphere are used to model non-spherical blocks, then the relative costs of HI can even decrease to less than ten percent of the total simulation time. This had been the case for our recently published simulation of a small peptide [17].

The third example demonstrates how constant density boundary conditions [16] can be used to model non-equilibrium transport scenarios with the brownmove package. Here, a diffusion current of the small mobile particles across an array of fixed spherical obstacles developed. This can be seen as a greatly simplified model of diffusive transport in a cell where various fixed structures like vesicles or the cytosceleton obstruct free diffusion. As expected, the resulting diffusion current decreased with increasing size and number of the obstacles. Interestingly, when an additional attractive interaction was added between the mobile particles and the obstacles, the diffusional transport was much less affected, because now the mobile "proteins" were guided along the surfaces of the obstacles through the narrow holes between them. One can speculate that a certain "stickiness" between the proteins and the structural elements of a cell might actually help with the efficient transport and thus at least partly compensate for blocking the direct path. In our example the mobile "proteins" and the obstacles were extremely simplified. However, it is straightforward to build much more realistic models of both the proteins and the cellular structures in brownmove by using multiple van-der-Waals spheres per bead or by adding any number of point charges to both the proteins and the obstacles. A more realistic fibrillar structure of the obstacles could for example be implemented by many small spheres along straight or curves lines within the simulation volume. Also crowding could be included by adding a fixed number of mobile particles of another species which then are not exchanged at the reservoirs.

In the last example we simulated a scenario where particles with a "sticky" patch formed temporary agglomerates. Usually such simulations with their fast and frequent association and dissociation events are tedious to analyse. Following a recent project [47], we mapped the spatial positions onto a dynamic interaction network where each particle is represented by a node and a link is added when the attractive patches of two particles are closer to each other than a specified minimal distance. From this dynamic network we then extracted the distribution of cluster sizes, the average and maximum number of links per node, and the number and average size of the clusters of at least two particles. Together with images from only two snapshots these time dependent network measures allowed to obtain a quantitative picture of the dynamics during the simulation.

The examples presented here are rather templates than actual projects. However, they not only emphasise the importance of HI but they also demonstrate that our freely available "brownmove" simulation package is very flexible and allows to easily investigate a number of scenarios for which a specialised software had to be written before. This especially applies to many-particle simulations with different kinds of particles in "open" systems where, e.g., non-equilibrium transport or reactions, or the association of particles from a large bulk phase onto a surface are considered.

## Methods

### Particle setup and interactions

As mentioned above, the particles in a "brownmove" simulation are set up hierarchically from various "objects" (see Figure 1). The "brownmove" package is written in C++ and each of these "objects" is implemented as a class. This design allows to easily extend the current protein model by new interaction types or new functionalities. In addition to the moving particles also fixed objects can be defined which interact with the mobile particles. These fixed objects are used to implement the walls of the simulation box or any rigid structures within this volume.

To model a particle, first a "Protein" object is defined. When this Protein object describes a rigid particle like a folded protein or a colloidal particle then it contains a single "Gestalt" object, which in turn contains the "Shape" objects that are responsible for the interactions. To implement a bead-spring polymer or a flexible protein, the "Protein" object contains multiple "Gestalt" objects and the definitions of the springs between these Gestalten. With this local definition of the connecting springs a single template molecule can be set up from which then multiple copies are drawn and inserted into the simulation during runtime. A "Protein" object thus defines an entity which is inserted or removed from a simulation as a whole.

The next level in the model hierarchy are the independently moving "Gestalt" objects. They keep track of their position and orientation and contain the "Shape" objects. The "GeomShape" is always present. It handles the generation of the random forces and then converts the total force accumulated during the current timestep into the corresponding displacement. Here the Langevin and the Brownian Dynamics propagation schemes are implemented. The other Shape objects model the physical interactions with the other particles, that is, during the force evaluation every pair of "Gestalt" objects compares whether they have Shapes of the same type. Then these two Shapes calculate their contribution to the mutual interaction.

Currently, five types of interactions are implemented. These are shielded Coulombic interactions between sets of point charges in the "EstatShape", effective short range van-der-Waals type interactions in the "vdWShape", bonds from harmonic and quartic terms in the "BondShape", external forces via the "ExternalShape", and hydrodynamic interactions via the "HIShape" objects.

Electrostatic interactions are implemented in a Debye-Hückel model via point charges which interact via a screened Coulomb potential Φ_ik_[49]. For a pair of charges *q_i_*and *q_k_*located on different "EstatShapes" the interaction energy is(2)

Here, ε is the relative dielectric constant of water and ε_0 _the vacuum dielectric constant. The shielding from ions in the solvent is captured in the inverse Debye length κ= 1/*l*_D_. In most cases, the point charges are embedded in the proteins where there are no counter ions to shield the interaction. This is accounted for by *B_ik_*= *b_i_*+ *b_k_*, the sum of the effective burial depths *b_i_*and *b_k_*. The point charges are defined in an internal coordinate system of the "EstatShape", which in turn can be placed at an arbitrary position and orientation into the coordinate system of the enclosing "Gestalt".

In brownmove the short range interactions between the surfaces of the proteins or colloidal particles are modelled phenomenologically. For this, an arbitrary number of so-called van-der-Waals spheres can be defined in the "vdWShape" objects, which then interact via a Lennard-Jones-type potential depending on the closest distance *r*_12 _between their surfaces.(3)

For each van-der-Waals sphere a position within the "vdWShape", a radius, and a "colour" index are specified. For each pair of colours different interaction parameters can be specified to model, e.g., short-range hydrophobic attractions or purely repulsive hydrophilic interaction patches. For numerical stability, the diverging Lennard-Jones interaction can be linearized when the spheres overlap more than a specified distance.

Bonds between "Gestalt" objects of the same "Protein" can be hooked up at arbitrary positions on the "BondShapes". This means that bonds are not restricted to the centers of a "Gestalt" but that they can be attached eccentrically. Currently, harmonic and quartic terms are implemented for the bond potentials.

Similar "hooks" for externally specified forces are provided by the "ExternalShape" objects. Currently implemented are a harmonic potential which allows to confine the position of a "Gestalt" to a certain position, a constant vectorial force which can be used to model, e.g., gravitational forces or a constant fluid velocity, and a shear field.

### Langevin and Brownian propagation schemes

To derive the implicit solvent propagation scheme for Brownian and Langevin Dynamics simulation, we start from Newton's equations of motion for the full system of the large proteins or colloidal particles and the many small solvent molecules. In this equation the forces *F_i_*on particle *i*, which lead to a change of its velocity *v_i_*, stem from the two-body interactions with all other particles.(4)

Here, *mi *is the mass of particle *i *and all pairwise forces are assumed to be conservative, i.e., they are the derivatives of an energy landscape depending on mutual distances. When we are interested in the motion of only the larger particles, the many explicitly considered solvent molecules can be replaced, as Einstein suggested, by a mean-field heat bath consisting of a Stokesian friction term *F*_r _= -γ*v *with the friction coefficient γ, and random kicks from the thermal motion of the solvent molecules. Their exact form is not known but it is sufficient to know that their average should vanish for an isotropic system and that they have an average temperature dependent strength. This is conveniently expressed via the statistical moments of the resulting displacements *R_i_*over a time interval Δ*t*:(5)

Here the indices *i *and *k *denote coordinates and the coupling between the coordinates via the displaced solvent is given by the diffusion tensor (D_ik_) which has 3*N *× 3*N *entries when only the translation of the *N *particles is considered. For a simulation it is more convenient to express the solvent induced random displacements in terms of effective forces *f*_i _which lead to the same displacements. With the relation between the friction coefficient γ_i _and the self diffusion coefficient *D*_ii _= k_B_T/γ_i _we get(6)

Then the many-particle Newton equation (4) reduces to a Langevin equation with a friction term and the effective forces *F_i _*= Σ *F_ik_*+ *f_i_*which are the sum of the external and the random forces.(7)

Assuming that the force *F*_i _remains constant during a short time interval *t *this equation can be integrated analytically to give the velocity *v*(Δ*t*) and the displacement Δ*x*(Δ*t*) at the end of the timestep Δ*t *when the initial velocity at the beginning of the time interval was *v*_0_. For convenience we drop the coordinate index *i *for the following.(8)(9)

These two equations can now directly be used to propagate the particles in the implicit-solvent approximation. This Langevin Dynamics (LD) propagation scheme assumes that the solvent can be substituted by time averaged random kicks and Stokesian friction and that Δ*t *is so small that the external force remains essentially constant when the particle is displaced by Δ*x*(Δ*t*). When the time step Δ*t *in the above equations (8) and (9) is much larger than the so-called velocity relaxation time τ_rel _= *m*/γ, the exponentials vanish and the LD propagation reduces to the standard Brownian Dynamics (BD) scheme(10)

where the velocity follows the force instantaneously and the displacement due to the external forces increases linearly with the timestep Δ*t*.

While in practical applications both for LD and BD there is the usual upper limit for the integration timestep where the numerical accuracy deteriorates, there is also a conceptual lower limit for Δ*t *in the BD approximation. As a rough estimate, Δ*t *should not be smaller than about ten times the velocity relaxation time τ_rel_. For BD simulations with a single particle type one usually finds an integration timestep which is large enough to be conceptually usable and also short enough for a numerically stable propagation, but this may not work any more when proteins of different sizes are considered. Then, a timestep which is stable enough for the faster smaller particles may be unphysically short for the slower larger ones. For more details on this problem see reference [17].

In brownmove both algorithms are implemented and can even be used within the same simulation. When a mass is defined for a given particle then the LD propagation scheme is used by the "GeomShape" object, otherwise the BD algorithm. Even though the LD equations of motion (8) and (9) look more complicated than the simple BD equations (10), the additional numerical costs are negligible, because most of the terms are constants and the effort for the actual propagation step scales linearly with the number of particles, while the evaluation of the pairwise forces scales quadratically. We therefore advocate to always use the LD propagation scheme for which only the easily controllable numerical accuracy puts a constraint on the timestep.

### Fast Hydrodynamics

Hydrodynamic interactions, which describe the coupling of the particle velocities via the displaced solvent, are modelled in "brownmove" via the Rotne-Prager-Yamakawa (RPY) tensor extended to handle rotation and particles of different radii [21-23,25,26]. There is no theoretically rigid formulation for hydrodynamic interactions of overlapping spheres of different sizes. However, a working ansatz was proposed by Durchschlag and Zipper [50]. For practical applications the extended RPY HI-tensor can handle a slight overlap of the beads before the results become numerically unstable. Consequently, in brownmove projects in which the hydrodynamic interaction between different physical particles is defined by their outer surface, any HIShape should be accompanied by a VdwShape with a repulsive short ranged potential to prevent the particles to come closer than their actual, hydrodynamically relevant radii.

The effective hydrodynamically corrected external forces *F*^eff ^acting on particle *i *are given by(11)

Applying the hydrodynamic coupling to the random forces is not that straightforward due to "temperature conservation"--the self diffusion of the particles which is a measure for their temperature is, for lower concentrations, not affected by the hydrodynamic interactions [51]. Consequently, the random forces have to be correlated with the square root of the diffusion tensor, see equation (5). The conventional Ermak-McCammon algorithm [2] uses a numerically expensive Cholesky factorisation for this. A numerically more efficient approach is the Chebychev approximation suggested by Fixman [30]. Other approaches which work well for special cases are the mean-field ansatz of Heyes [8] or the accelerated Stokesian dynamics of Banchio and Brady [31]. An even faster approximation could be derived by us by proposing effective HI correlated random forces similar to equation (5). To account for the square root of the diffusion tensor, expansion coefficients are used in the spirit of a Taylor series.(12)

The normalisation factors *C*_i _and the weights *β*_ik _can be determined approximately and the resulting truncated expansion approximation hydrodynamics (TEA-HI) recovers at least 90% of the correlations at a runtime scaling which increases only quadratically with the particle number [18]. With this approximation the importance of hydrodynamic interactions can now be investigated for all those systems for which the also quadratically increasing runtime for the evaluation of the pairwise interactions is possible. With *β*_ii _= 1 the normalisation factors of equation (12) can be determined from(13)

and the quadratic equation(14)

where ε = <*D*_ik _/*D*_ii_> is the average of the normalised off-diagonal entries of the diffusion matrix. Apart from the faster evaluation, this approximate form of the HI has the advantage that the sums in equations (12), (13), and (14) can be evaluated simultaneously with very low memory requirements from the temporarily set up two-body submatrices of the diffusion tensor. With this even large many-particle simulations fit into the fast level-3 cache of current CPUs.

In "brownmove" the above TEA-HI algorithm is implemented in the "HiShape" objects in which currently a single hydrodynamic sphere can be defined. How the hydrodynamic coupling, which is based on an instantaneous flow field, can be combined with the LD algorithm is explained in detail in reference [17]. The basic idea is to apply the HI correlations to average forces, which would lead to the same displacements during the timestep in the BD picture, i.e., when the acceleration is ignored.

Coming back to the problem of bead overlap, in the original algorithm of Ermak and McCammon the results degrade with increasing overlap because the RPY tensor does not cover this regime whereas with Fixman's Chebychev approximation additionally the convergence becomes slower due to the diverging range of the eigenvectors when the spheres start to overlap [52]. This then requires more terms for the approximation to converge to the specified accuracy. On the other hand, our TEA-HI approximation resembles a Taylor expansion that is always truncated after the first correction term regardless of the achieved accuracy. Consequently, the runtime is not affected by the particle separation. As detailed in [18], the truncation errors increase up to 5% in a dimer of touching spheres which is less than the errors involved in the long-range expansion RPY tensor. Consequently, with a diffusion tensor that correctly describes near-field HI, our method would lead to a relative error of up to 5-10% for the pairs of close particles and less for all the further separated pairs of particles in the simulation.

### Boundary conditions

In "brownmove" various boundary condition can be used. The most simple scenario is an infinite simulation volume which would be used, e.g., to verify the long time diffusional behaviour of a flexible protein assembled from multiple sub-units. To confine the particles, "brownmove" allows to specify combinations of simple reflecting walls, one, two, or three dimensional periodic setups, and walls with a "Gestalt". By defining a "Gestalt" for a wall, not only planar van-der-Waals surfaces can be defined but also static structures like membrane proteins built from van-der-Waals spheres and point charges. A "Wall" object can thus also be used to model a rigid network of microtubili or non-mobile vesicles around which the proteins have to find their way. When periodic boundary conditions are specified, "brownmove" uses image particles during the evaluation of the interactions.

A special type of boundary condition is implemented with the use of particle acceptors and injectors, which allow to define constant density reservoirs and implement reactions at a membrane. The idea of a constant density interface is the following [16]. When a large simulation volume is divided by a virtual wall then particles will cross this boundary with an average rate that depends on their density and their diffusion coefficient. Now the volume on the other side of the virtual wall can be omitted when all particles that cross from the cis to the trans side are removed from the simulation and at the same time new particles are inserted randomly close to the interface with a rate and distribution that corresponds to the assumed density on the trans side. When the density on the trans side is higher more particles will be inserted into the simulation than leave until the density inside the simulation equals the density specified at the interface. Such an interface is especially useful for simulations where the adsorption of particles to surfaces is studied [11]. Here, the constant density interface behaves like an infinitely large bulk and the density above the surface will remain constant no matter how many particles bind. The same algorithm can also be used to model finite reservoirs or non-equilibrium conditions that lead to a diffusion current through the simulation volume. By taking out one type of particles and inserting a different type at the same position reactions like charge transfer can be modeled. More details are given in reference [16].

### Data analysis

For maximal flexibility the output of a brownmove simulation is not directly saved to disk, which would often result in unnecessarily large output files. The particle positions are rather piped into a command that is specified in the setup file. In the most simple case this command dumps the particle positions to a file. If, e.g., from a simulation of a bead-spring polymer only the center of mass and the vector from the first to the last bead is required at each output interval, the output command would extract that information on the fly and only save the processed output to disk. The output commands can be simple scripts, full-fledged analysis tools, or even multiple analysis programs chained together via pipes.

### Availability

The brownmove simulation package which was presented here is freely available for academic use. The latest version can be downloaded together with documentation and some examples at http://service.bioinformatik.uni-saarland.de/brownmove.

## Authors' contributions

TG wrote the simulation and analysis software, conceived, performed, and analyzed the simulations, and wrote the manuscript.

## Supplementary Material

Additional file 1 **Brownmove definition file for a constrained bead-spring polymer**. This (ASCII text) file defines a bead-spring polymer for a brownmove simulation with five beads connected by springs between the direct and the next neighbours. Each bead has a single van-der-Waals sphere to prevent mutual overlap between the beads, a sphere for hydrodynamic interactions, and two to four hook-up points for the connecting springs. For further details see the comments in this protein definition file. Click here for file

Additional file 2 **Simulation setup file for the bead-spring polymer examples**. This (ASCII text) file defines the global parameters, which polymer to use, and the boundary conditions for the bead-spring polymer example simulations. For details see the comments in the file. Click here for file

Additional file 3 **Example of a bead-train polymer definition file**. This brownmove protein definition file gives an example for how a short bead-train polymer as sketched in Figure 2B can be defined in brownmove. It consists of three rotating beads which have a van-der-Waals sphere and off-center hooks for the springs between adjacent beads. For more details see the comments in the file. Click here for file

Additional file 4 **Brownmove definition file for the *N *= 13 "elastic protein"**. For more details see the comments in this (ASCII text) file. Click here for file

Additional file 5 **Simulation setup file for the diffusion-between-obstacles examples**. Brownmove simulation setup file for a 2D periodic box with two oppositely placed constant density interfaces and an array of obstacles as sketched in Figure 9. The constant density interfaces and the obstacles are defined in "boxWithObstacles.bdef" which is given as additional file 6, while the mobile particles are defined in "simpleBead.bdef" (see additional file 7). For more details see the comments in the setup file. Click here for file

Additional file 6 **Brownmove definition file for a simulation box with two constant density interfaces and a central array of fixed spherical obstacles (see Figure **9). For more details see the comments in this (ASCII text) file. Click here for file

Additional file 7 **Brownmove particle definition file for a minimal spherical, uncharged, van-der-Waals particle used in the diffusion-between-obstacles example**. Click here for file

Additional file 8 **Brownmove particle definition file for the agglomeration-with-network-analysis example simulation**. As sketched in Figure 10 A the particle consists of two displaced van-der-Waals spheres with different "colour" indices. Together with the parameter definitions in the simulation setup file (see additional file 9) the spheres with colour 0 can stick to each other while all other pairs have purely repulsive interactions. For more details see the comments in this (ASCII text) file. Click here for file

Additional file 9 **Brownmove simulation setup file for the agglomeration-with-network-analysis example simulation**. Click here for file

## References

[B1] Einstein A Über die von der molekularkinetischen Theorie der Wärme geforderte Bewegung von in ruhenden Flüssigkeiten suspendierten Teilchen Ann Phys 1905 17 549 560 10.1002/andp.19053220806

[B2] Ermak DL McCammon JA Brownian dynamics with hydrodynamic interactions J Chem Phys 1978 69 1352 1360 10.1063/1.436761

[B3] Elcock AH Gabdoulline RR Wade RC McCammon JA Computer simulation of protein-protein association kinetics: Acetylcholinesterase-fasciculin J Mol Biol 1999 291 149 162 10.1006/jmbi.1999.2919 10438612

[B4] Gabdoulline RR Wade RC Biomolecular diffusional association Curr Opin Struct Biol 2002 12 204 213 10.1016/S0959-440X(02)00311-1 11959498

[B5] Spaar A Floeck D Helms V Association of cytochrome *c *with membrane-bound cytochrome *c *oxidase proceeds parallel to the membrane rather than in bulk solution Biophys J 2009 96 1721 1732 10.1016/j.bpj.2008.11.052 19254533 PMC2717263

[B6] Harel M Spaar A Schreiber G Fruitful and futile encounters along the association reaction between proteins Biophys J 2009 96 4237 4248 10.1016/j.bpj.2009.02.054 19450494 PMC2712199

[B7] Dünweg B Reith D Steinhauser M Kremer K Corrections to scaling in the hydrodynamic properties of dilute polymer solutions J Chem Phys 2002 117 914 924

[B8] Heyes DM Mean-field hydrodynamics Brownian dynamics simulations of viscosity and self-diffusion of near-hard-sphere colloidal liquids J Phys: Condens Matter 1995 7 8857 8865 10.1088/0953-8984/7/47/006

[B9] McGuffee SR Elcock AH Diffusion, crowding & protein stability in a dynamic molecular model of the bacterial cytoplasm PLoS Comput Biol 2010 6 e1000694 10.1371/journal.pcbi.1000694 20221255 PMC2832674

[B10] Gorba C Helms V Diffusional dynamics of cytochrome c molecules in the presence of a charged surface Soft Materials 2003 1 187 204 10.1081/SMTS-120021736 15260567

[B11] Gorba C Geyer T Helms V Brownian dynamics simulations of simplified cytochrome c molecules in the presence of a charged membrane J Chem Phys 2004 121 457 464 10.1063/1.1755668 15260567

[B12] Gabdoulline RR Wade RC Brownian dynamics simulation of protein-protein encounter Methods 1998 3 329 341 10.1006/meth.1998.0588 9571088

[B13] Gabdoulline RR Wade RC Simulation of the diffusional association of barnase and barstar Biophys J 1997 72 1917 1929 10.1016/S0006-3495(97)78838-6 9129797 PMC1184389

[B14] Huber GA McCammon JA BrownDye: A software package for Brownian dynamics Comput Phys Comm 2010 181 1896 1905 10.1016/j.cpc.2010.07.022 PMC2994412 21132109

[B15] García de la Torre J Huertas M Carrasco B Calculation of hydrodynamic properties of globular proteins from their atomic-level structures Biophys J 2000 78 719 730 10653785 10.1016/S0006-3495(00)76630-6 PMC1300675

[B16] Geyer T Gorba C Helms V Interfacing Brownian dynamics simulations J Chem Phys 2004 120 4573 4580 10.1063/1.1647522 15267316

[B17] Winter U Geyer T Coarse grained simulations of a small peptide: Effects of finite damping and hydrodynamic interactions J Chem Phys 2009 131 104102 10.1063/1.3216573 19317564

[B18] Geyer T Winter U An O(*N*^2^) approximation for hydrodynamic interactions in Brownian dynamics simulations J Chem Phys 2009 130 114905 10.1063/1.3089668 19317564

[B19] Dhont JKG An introduction to dynamics of colloids 1996 Amsterdam, Elsevier

[B20] Kirkwood JG Riseman J The intrinsic viscosities and diffusion constants of flexible macromolecules in solution J Chem Phys 1948 16 565 573 10.1063/1.1746947

[B21] Rotne J Prager S Variational treatment of hydrodynamic interaction in polymers J Chem Phys 1969 50 4831 4837 10.1063/1.1670977

[B22] Yamakawa H Transport properties of polymer chains in dilute solutions: hydrodynamic interactions J Chem Phys 1970 53 436 443 10.1063/1.1673799

[B23] Dickinson E Allison SA McCammon JA Brownian dynamics with rotation-translation coupling J Chem Soc Faraday Trans 2 1985 81 591 601 10.1039/f29858100591

[B24] Mazur P van Saarloos W Many-sphere hydrodynamic interactions and mobilities in a suspension Physica 1982 115A 21 57

[B25] García de la Torre J Bloomfeld VA Hydrodynamic properties of macromolecular complexes. I. Translation Biopolymers 1977 16 1747 1763

[B26] García de la Torre J Bloomfeld VA Hydrodynamic properties of macromolecular complexes. II. Rotation Biopolymers 1977 16 1765 1778 10.1002/bip.1977.360160813 890069

[B27] Cichocki B Ekiel-Jezewska ML Wajnryb E Lubrication corrections for three-particle contributions to short-time self-diffusion coefficients in colloidal dispersions J Chem Phys 1999 111 3265 3273 10.1063/1.479605

[B28] García de la Torre J Bloomfield VA Hydrodynamics of macromolecular complexes. III. Bacterial viruses Biopolymers 1977 16 1779 1793 890069 10.1002/bip.1977.360160813

[B29] Zipper P Durchschlag H Hydrodynamic multibead modelling: problems, pitfalls, and solutions. 1. Ellipsoid models Eur Biophys J 2010 39 437 447 10.1007/s00249-009-0424-2 19280183

[B30] Fixman M Construction of Langevin forces in the simulation of hydrodynamic interaction Macromolecules 1986 19 1204 1207 10.1021/ma00158a043

[B31] Banchio AJ Brady JF Accelerated Stokesian dynamics: Brownian motion J Chem Phys 2003 118 10323 10332 10.1063/1.1571819

[B32] Tanaka H Araki T Simulation method of colloidal suspensions with hydrodynamic interactions: Fluid particle dynamics Phys Rev Lett 2000 85 1338 1341 10.1103/PhysRevLett.85.1338 10991546

[B33] Nakayama Y Yamamoto R Simulation method to resolve hydrodynamic interactions in colloidal dispersions Phys Rev E 2005 71 036707 10.1103/PhysRevE.71.036707 15903633

[B34] Hoogerbrugge PJ Koelman JMVA Simulating microscopic hydrodynamic phenomena with dissipative particle dynamics Europhys Lett 1992 19 155 160 10.1209/0295-5075/19/3/001

[B35] Español P Warren P Statistical mechanics of dissipative particle dynamics Europhys Lett 1995 30 191 196

[B36] Malevanets A Kapral R Mesoscopic model for solvent dynamics J Chem Phys 1999 110 8605 8613 10.1063/1.478857

[B37] Gompper G Ihle T Kroll DM Winkler RG Multi-particle collision dynamics: A particle-based mesoscale simulation approach to the hydrodynamics of complex fluids Adv Polymer Science 2009 221 1 87

[B38] Lowe CP An alternative approach to dissipative particle dynamics Europhys Lett 1999 47 145 151 10.1209/epl/i1999-00365-x

[B39] Chen S Doolen GD Lattice Boltzmann method for fluid flows Annu Rev Fluid Mech 1998 30 329 364 10.1146/annurev.fluid.30.1.329

[B40] Ahlrichs P Everaers R Dünweg B Screening of hydrodynamic interactions in semidilute polymer solutions: A computer simulation study Phys Rev E 2001 64 040501(R) 10.1103/PhysRevE.64.040501 11689999

[B41] Northrup SH Reynolds JCL Miller CM Forrest KJ Boles JO Diffusion-controlled association rate of cytochrome *c *and cytochrome *c *peroxidase in a simple electrostatic model J Am Chem Soc 1986 108 8162 8170 10.1021/ja00286a008

[B42] Li B Madras N Sokol AD Critical exponents, hyperscaling, and universal amplitude ratios for two- and three-dimensional self-avoiding walks J Stat Phys 1995 80 661 754 10.1007/BF02178552

[B43] García de la Torre J Amorós D Ortega A Intrinsic viscosity of bead models for macromolecules and nanoparticles Eur Biophys J 2010 39 381 388 19198827 10.1007/s00249-009-0405-5

[B44] Frembgen-Kesner T Elcock AH Absolute protein-protein association rate constants from flexible, coarse-grained Brownian dynamics simulations: The role of intermolecular hydrodynamic interactions in barnase-barstar association Biophys J 2010 99 L75 L77 10.1016/j.bpj.2010.09.006 21044566 PMC2965997

[B45] Frembgen-Kesner T Elcock AH Striking effects of hydrodynamic interactions on the simulated diffusion and folding of proteins J Chem Theory Comput 2009 5 242 256 10.1021/ct800499p 26610102

[B46] Sholl DS Fenwick MK Atman E Prieve DC Brownian dynamics simulation of the motion of a rigid sphere in a viscous fluid very near a wall J Chem Phys 2000 113 9268 9278 10.1063/1.1320829

[B47] Lauck F Helms V Geyer T Graph measures reveal fine structure of complexes forming in multiparticle simulations J Chem Theory Comput 2009 5 641 648 10.1021/ct800396v 26610228

[B48] Helms V Principles of computational cell biology 2008 Weinheim, Wiley-VCH

[B49] Medina-Noyola M McQuarrie DA On the interaction of spherical double layers J Chem Phys 1980 73 6279 6283 10.1063/1.440125

[B50] Carrasco B García de la Torre J Zipper P Calculation of hydrodynamic properties of macromolecular bead models with overlapping spheres Eur Biophys J 1999 28 510 515 10.1007/s002490050233 10460344

[B51] Deutch JM Oppenheim I Molecular theory of Brownian motion for several particles J Chem Phys 1971 54 3547 3555 10.1063/1.1675379

[B52] Jendrejack RM Graham MD de Pablo JJ Hydrodynamic interactions in long chain polymers: Application of the Chebyshev polynomial approximation in stochastic simulations J Chem Phys 2000 113 2894 2900 10.1063/1.1305884

